# World’s Hom. Convention of 1876

**Published:** 1881-04

**Authors:** 


Transactions of the world's homœopathic convention of 1876, vol. ii., historical. Edited by J. C. Guernsey, M. D. American Inst. of Homœopathy.

This volume is said to contain historical sketches of homœopathy in all climes, with a bibliographical resume. We hope the history it gives of other sections is more correct than that of Philadelphia; ample proof of errors in this portion will be furnished anon. The editor desires that all members of the Institute who have failed to receive this volume will notify him.

